# Effect of Anti-Obesity and Antioxidant Activity through the Additional Consumption of Peel from ‘Fuji’ Pre-Washed Apple

**DOI:** 10.3390/foods11040497

**Published:** 2022-02-09

**Authors:** Da-Yeong Ko, Kang-Mo Ku

**Affiliations:** 1 Department of Horticulture, College of Agriculture and Life Sciences, Chonnam National University, Gwangju 61186, Korea; ko.dayeong1996@gmail.com; 2 Interdisciplinary Program in IT-Bio Convergence System, Chonnam National University, Gwangju 61186, Korea

**Keywords:** anti-obesity, apple peel, fresh-cut foods, 3T3-L1, pre-washed apple

## Abstract

There is limited information on the health effects of apple peel taken from ‘Fuji’ (*Malus pumila* Mill) apples washed with ozonated water. To clarify the health-promoting effects of peel, the triterpenoids (ursolic acid and oleanolic acid) were quantified with gas chromatograph–mass spectrometry. Anti-obesity effects of apple peel extract on the 3T3-L1 pre-adipocyte cell were compared with apple flesh, whole apple, and ursolic acid. The peel extract treatment with 3.30 ± 1.05 μM of ursolic acid significantly suppressed (*p* < 0.05) the lipid accumulation compared with the content in flesh, and a similar level was reached in the 5 μM ursolic acid positive control group. In the peel extract and ursolic acid treatment groups, the C16:0 concentration was significantly inhibited (*p* < 0.05), implying the anti-obesity effect of ursolic acid on the 3T3-L1 cell. Moreover, apple peel contributed 41% of the total flavonoids content and 31% of the phenolic contents of the whole apple, but only accounted for less than 10% of the whole apple (weight basis). This study’s results offer basic data on pre-washed apple as a health functional food, offering information about the health benefits of apple peel, calculated based on the partial ratio in the whole apple.

## 1. Introduction

According to the World Health Organization (WHO), obesity in modern society is defined by abnormal or excessive lipid accumulation [1]. The prevalence of obesity is on the rise internationally, meaning social costs are also increasing due to obesity causing diseases such as fatty liver, cardiovascular high blood pleasure, and diabetes [2]. Reducing obesity is becoming an important social issue, and ongoing research is being conducted aiming to decrease overall health problems using health-promoting compounds derived from natural sources [3]. Attempts to address obesity using drugs derived from natural sources have shown success in several cases, reducing lipid accumulation in the body [4]. Compounds derived from apples, such as minerals and amino acids, actively engage in the body’s metabolic activities. Moreover, antioxidant compounds in apples, such as phenolic compounds and flavonoids, and other bio-active compounds, especially ursolic acid, have a therapeutic effect [5,6]. Moreover, the WHO also suggested that consuming more than 400 g of fruits, vegetables and nuts per day could reduce the risk of various diseases [7].

Despite the recommendations of the WHO, the U.S. dietary surveillance study reported that most of the population do not eat the recommended daily amount of fresh fruit and vegetables [8]. Likewise, in 2016, National Health Statistics given by Disease Control and Prevention in South Korea reported that only 37.9% of people achieve the recommend intakes [9].

Recently, in regard to this intake habit of consumers, the fresh-cut food market has been growing [10]. Fresh-cut foods allow consumers to consume fruit and vegetables without the inconvenience of washing, peeling, and slicing. The ‘Fuji’ apple (*Malus pumila* Mill), one of the major apple cultivars in South Korea, is also sold as a fresh-cut product. Apples are historically known as healthy foods [11]. The microorganisms or residual pesticides on the surfaces of fruits and vegetables that cause food poisoning or health issues can be removed via ozonized water washing [12,13]. Apples are a rich source of antioxidant compounds, and their peel contains more antioxidant compounds than their flesh by weight [14]. The antioxidant compounds in apple peel are mostly polyphenols and flavonoids, and its anti-obesity effects have been reported [15]. The triterpenoid found in apple is mainly represented by ursolic acid, which helps weight loss by suppressing triglyceride accumulation in blood vessels, thus lowering the incidence of high blood pressure and diabetes, as well as cardiovascular risk [16].

In previous research, ursolic acid has been reported to reduce triglyceride accumulation in 3T3-L1 cells, which are widely used as a white fat cell model [17]. A low triglyceride accumulation tendency has also been shown in relation to apple pomace [18]. This result suggests that the apple waste, or pomace (mostly peel and cell wall) reduces triglyceride levels, resulting in an anti-obesity effect. Anti-obesity effect of apples has also been proven through another previous research [19,20]. Although the anti-obesity effects of ordinary “Fuji” apples have already been explored, the health contributions of apple peel from actual pre-washed apples have not been intensively investigated. Moreover, the fatty acid metabolism changes caused by health-promoting compounds in apple have not been intensively studied using 3T3-L1 cells.

Thus, this study aims to offer basic data on pre-washed apple as a functional food by investing the additional health values of apple peel. Specifically, the anti-obesity effect of each apple tissue (whole apple, flesh, and peel) will be calculated based on their weight ratio in pre-washed whole apples. The lipid and fatty acid synthesis suppression effects on the 3T3-L1 cells by different apple tissue extracts will be discussed.

## 2. Materials and Methods

### 2.1. Plant Material Preparation and Water Contents Calculation

For the experiments, pre-washed ‘Fuji’ (*Malus pumila* Mill) apples were bought at the local market. The pre-washed apples had been through 3 steps of washing, which were flooding, brushing with ozonized water, and full drying with air and far-infrared light at the Uiseong agricultural products processing center (APC). Five apples were randomly grouped in one biological replication and three groups were prepared. Each apple was peeled uniformly using a Jonny Apple Peeler (“Apple Slicer, Corer, Peeler and Pie Maker VKP1010”, VKP Brands, Orem, UT, USA). Each sample of separated peel and flesh (without seed and core) was lyophilized at 5 mTorr and −85 °C (FD8508, IlShinBioBase, Dongducheon, Korea). After complete drying in the freeze-drier, each biological replication was ground with a commercial coffee grinder (BCG-620SP, Bean Cruise, Seoul, Korea) and filtered through a standard sieve (No. 18.50, 300 μm, 1 mm, Chung Gye Sang Gong Sa, Seoul, Korea). The dried powder was stored at −20 °C. The fresh weight (FW) and dry weight [1] of the separated apple peel and flesh were measured before and after freeze-drying, so that the water contents and proportions of peel and flesh could be calculated. The proportions in each tissue were used to prepare whole-apple (peel + flesh) treatments following sample extraction. The water content information was used for converting DW to FW 100 g. In order to determine the water contents, we used the weight difference between FW and DW following oven-drying [21].

The water contents of the apple samples were calculated using the following equation:

(1) $$\mathrm{Water}\;\mathrm{contents}\;\left(\%\right)=\frac{\mathrm{FW}-\mathrm{DW}}{\mathrm{FW}}\times 100$$

### 2.2. Antioxidant Assays and Antioxidant Compounds

#### 2.2.1. Sample Extraction for Antioxidant Assays and Compounds

Apple sample extraction for antioxidant measurement was performed in the following manner: 50 mg of dried powder was extracted with 1 mL of 70% methanol. The mixture was incubated at 90 °C for 10 min and centrifuged at 10,000× *g*. The supernatant was moved into another vial and stored at 4 °C. All the antioxidant determination assays were performed within one week.

#### 2.2.2. 2,2-Diphenyl-1-picrylhydrazyl (DPPH) Radical Scavenging Activity

DPPH scavenging activity was analysed following the method developed by Ku and Kang [22]. A DPPH assay was performed to estimate the antioxidant capacity of the samples. To make a DPPH solution, 8 mg of DPPH (Sigma Aldrich Inc., Saint Louis, MO, USA) was dissolved in 50 mL methanol 80% with the optical density adjusted to 1.00 for reproducibility. Then, 10 μL of sample extract or positive control (0.01–0.5 mg·mL^−1^ gallic acid) was mixed with 190 μL DPPH solution in a 96-well plate and left to react at room temperature for 30 min. The absorbance of the reacted mixture was measured at 517 nm. The result has been expressed as gallic acid equivalent concentration (mg·100 g^−1^ FW).

#### 2.2.3. Ferric Reducing Antioxidant Power Assay (FRAP)

This assay used for apple extractions followed the original method described by Benzie and Strain [23]. The FRAP reagent was freshly made before the experiments by blending 30 mL of acetic acid buffer (pH3.0) and 3 mL of 10 mM 2,4,6-tri(2-pyridyl)-s-triazine (Sigma Aldrich Inc.) in 40 mM HCl, and 3 mL of freshly prepared 20 mM FeCl_3_ (Sigma Aldrich Inc.) in distilled water. The FRAP reagents were heated at 37 °C. Then, 10 μL of sample extract or positive control (0.02–0.25 mg·mL^−1^ gallic acid) and 300 μL of FRAP reagent were mixed in the 96-well plates. After 8 min of reaction, the samples were measured at 593 nm. The result has been expressed as gallic acid equivalent concentration (mg·100 g^−1^ FW).

#### 2.2.4. Total Flavonoid Content Assay (TFC)

The total flavonoids were measured via the following method: 20 μL of sample extract or standard (0.007–1 mg·mL^−1^ naringin) was mixed with 160 μL diethylene glycol in 96-well plates. The mixture was incubated for 5 min at room temperature and 10 μL of 0.5 N NaOH was added. After 30 min of reaction, the reacted samples were measured at 415 nm. The result has been expressed as naringin equivalent (NAE) concentration (mg·100 g^−1^ FW).

#### 2.2.5. Total Polyphenol Content Assay (TPC)

The polyphenol compounds in apple extracts were determined using the Folin–Ciocalteu method modified by Ku and Kang [22], whereby 100 µL of 0.2 M Folin–Ciocalteu’s polyphenol reagent (Sigma Aldrich Inc.) was mixed in a 96-well plate with 10 µL of sample extract or positive control (0.007–1 mg·mL^−1^ gallic acid). After reacting at room temperature for 3 min, 100 μL of 7.5% Na_2_CO_3_ (Sigma Aldrich Inc.) was added and incubated at room temperature for 30 min more. The absorbance was measured at 715 nm. The result has been expressed as gallic acid equivalent (GAE) concentration (mg·100 g^−1^ FW).

### 2.3. Triterpenoid Determination

#### 2.3.1. Triterpenoid Extraction and Concentrated Extract Sample Preparation

One gram of dried powder was extracted twice with 20 mL of 70% methanol in water bath (WB-11, DAIHAN Scientific Co., Ltd., Wonju-Shi, Korea) at 90 °C for 10 min each. Both supernatants were collected after being centrifuged for 3 min at 10,000× g. A 36 mL volume of extraction fluid was transferred to glass conical tubes, and 18 mL of CHCl3 was added and vortexed vigorously. Then, for extracting organic soluble compounds, 15 mL of distilled water was added. The mixtures were vortexed for 30 s and centrifuged at 4000× g for 3 min. After the two layers were separated, 16 mL of organic phase was moved into a glass conical tube, and the solvent was dried out under a nitrogen stream. A 200 μL volume of dimethyl sulfoxide (DMSO, Sigma Aldrich Inc.) was added, and the dry residue was dissolved completely. The final concentration of each sample was adjusted to 4 g DW∙mL^−1^. For the following cell culture assay, the cell cytotoxicity for 3T3-L1 cells was determined. According to the cell cytotoxicity assay (WST-1) results, concentrated samples were diluted to a concentration representing more than 90% cell viability. Therefore, 1 mg DW∙mL^−1^ of concentrated sample extract and 5 μM ursolic acid (positive control) were used in the cell culture assay.

#### 2.3.2. Sample Derivatization for Quantification

A 400 μL volume of the above CHCl_3_ layer was moved into reactive vials (Thermo fisher, Waltham, MA, USA). The solvent was completely evaporated under a nitrogen stream. The derivatization process was performed according to a modified method examined by [24]. A total of 250 μL of freshly made derivatization reagent made with N, O-Bis(trimethylsilyl)trifluoroacetamide (BSTFA) and pyridine (35:65 *v*/*v*) was added to the dry residue. The samples were incubated at 30 °C for 2 h. After incubation, 750 μL of hexane was added and analyzed with a gas chromatograph mass-spectrometer detector (GCMS-QP2020 NX, Shimadzu, Kyoto, Japan) fused column DB-5 (30 m, 0.25 mm, 0.25 µm, Agilent Technologies, Santa Clara, CA, USA). Authentic standards, including ursolic acid and oleanolic acid (Sigma Aldrich Inc.), were prepared through the same steps and used for external standard quantification.

### 2.4. Cell Culture

#### 2.4.1. Cell Plate Preparation

Dulbecco’s modified Eagle’s medium (DMEM, Gibco, Grand Island, New York, NY, USA), to which was added 100 μg·mL^−1^ penicillin and 100 μg·mL^−1^ streptomycin, was used for growing mouse 3T3-L1 cells (CCL-92.1, American Type Culture Collection, Manassas, VA, USA). During cell culture, the cells were incubated in a cabinet (MCO-170AC, Tokyo, Japan) at 37 °C in a humidified atmosphere of 5% CO_2_. A total of 10^5^ cells per well and 2 × 10^3^ per well were cultured in a 6-well plate and 96-well plate, respectively. A 10% concentration of calf serum (Gibco) was added into the growth medium, and the cells were incubated for 2 days until confluent. Nine 6-well plates for three different experiments (*n* = 3) and one 96-well plate for the WST-1 assay were prepared. For assessing lipid accumulation under different conditions, each sample of concentrated extract (1 mg DW∙mL^−1^) and a ursolic acid positive control (5 μM) were added to differentiation medium, which was DMEM containing 10% fetal bovine serum (FBS), 10 μg·mL^−1^ insulin, 0.5 mM isobutyl methylxanthine and 0.1 μM dexamethasone, for 48 h. The medium was replaced every two days.

#### 2.4.2. WST-1 Assay

The cell cytotoxicity of the concentrated apple extracts and the ursolic acid was determined on a 96-well cell culture plate using a WST-1 kit (EZ-3000, DuGenBio, Seoul, Korea). The result of the cytotoxicity analysis has been expressed as a percentage of the control.

#### 2.4.3. Oil-Red O Assay

At 9 days after differentiation, an Oil Red-O assay [25] was carried out to detect the presence of intercellular lipids in the 3T3-L1 cells. All the growth media were discarded first without vacuum pump suction. The cells were washed twice with PBS and fixed with a 3.5% formalin-containing PBS solution at 4 °C for 1 h. The fixed cells were washed twice with distilled water. Then, 1 mL of 0.3% Oil Red-O (in 60% isopropanol) solution was added and incubated for 15 min at room temperature. The Oil Red-O solution was washed gently with distilled water until the excess residue was removed. The stained cell plate was observed with a microscope (× 400). Stained lipid droplets in the 3T3-L1 cells were extracted with isopropanol, and then the absorbance of the extracts was measured at 490 nm.

#### 2.4.4. Cell Lipid and Fatty Acid Quantification

Total triglyceride (TG) and fatty acids (FA) were assessed in parallel after harvesting the cells (*n* = 3). At 9 days after differentiation, the cells were harvested with 0.25% trypsin-ethylenediaminetetraacetic (Gibco) and washed with PBS twice. The total TG contents were measured using an EZ-Triglyceride quantification assay kit (DG-TGC100, DugenBio, Seoul, Korea). Meanwhile, using the other harvested cells, the cellular lipids were extracted via a modified version of the procedure developed by Bligh and Dyer [26]. The cell pellets were extracted with 500 μL of chloroform with an internal standard of methyl heptadecanoate (5 μg·mL^−1^) and 250 μL of methanol. The mixtures were vortexed intensely and sonicated at 30 Hz for 30 min at 4 °C. Then, 250 μL of methanol with 250 μL of water was added before sonication for 10 min more. The mixture was centrifuged at 3000× *g* for 3 min. Then, 300 μL of organic phase was moved to a reactive vial. All the solvent was dried under N_2_ gas. Fatty acid methyl ester (FAME) assessment was performed, during which 100 μL of 0.5 N methanolic NaOH was added and heated at 90 °C for 15 min, and then we added another 500 μL. The samples were cooled for 5 min, then 500 μL of 14% boron trifluoride methanol solution (Sigma Aldrich Inc.) was added and the solution was vortexed. The samples were heated for 30 min at 90 °C and cooled. Lastly, 500 μL of hexane was added and shaken. Then, 200 μL from the upper layer (organic phase) was collected in a GC vial after centrifuging at 3000× *g*. Fatty acid methyl esters were quantified by a gas chromatograph mass-spectrometer detector (GCMS-QP2020 NX, Shimadzu, Kyoto, Japan) fused Column DB-5 (30 m, 0.25 mm, 0.25 µm, Agilent Technologies, Santa Clara, CA, USA). The samples were loaded onto the column via 3 split 1 μL injections. The initial oven temperature was 70 °C, which increased at 5 °C per min to 250 °C, and was then held for 1 min. Next, the temperature was increased at 20 °C per min to 310 °C and held for 10 min. The inlet and detector temperatures were maintained at 230 °C. The helium carrier gas flow rate through the column was 1.20 mL per min. Peaks in the chromatograms were identified using 37 compounds methyl ester standards (Sigma Aldrich Inc.) and quantified using internal standard methyl heptadecanoate (Sigma Aldrich Inc.). The results are reported as μg fatty acid per 10^5^ cells.

### 2.5. Statistical Analysis

To verify the results of this research, all experiments were carried out in triplicate, and the values are expressed as means ± standard deviation. After the analysis, Tukey’s HSD tests (*p* < 0.05) were run using SPSS 18.0 for Windows (SPSS Inc., Chicago, IL, USA).

## 3. Results and Discussion

### 3.1. Plant Material Preparation and Water Contents Calculation

**Figure 1:**
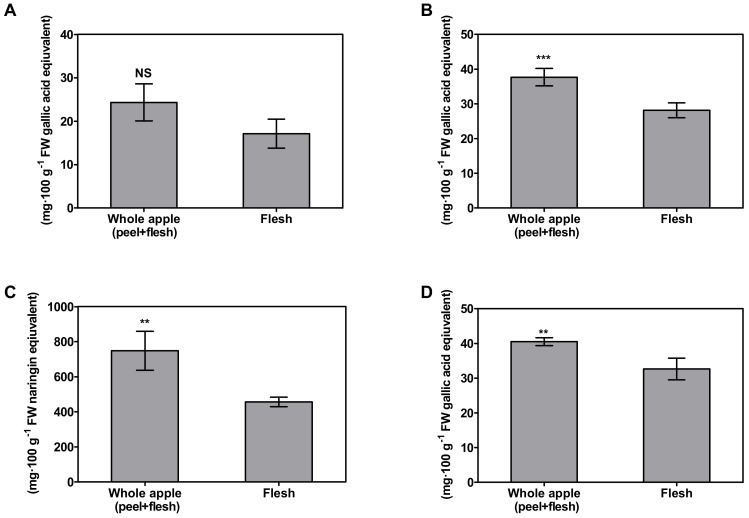
Antioxidant activities and compounds in different antioxidant assays comparing whole apple (peel + flesh) and flesh. NS, asterisk **, and asterisk *** indicate non-significant and significant differences between the peel and the flesh within the same assay, assessed by Student *t*-test at *p* > 0.05, *p* < 0.01, and *p* < 0.001, respectively. (**A**): Ferric-reducing antioxidant power; (**B**): DPPH radical scavenging activity; (**C**): total flavonoid contents; (**D**): total phenolic contents.

The benefits of peel were investigated in relation to the whole apple (peel + flesh) through the individual analyses of peel and flesh. For accurate measurement, the peel was uniform separated using a Jonny Apple Peeler. This experiment confirms that the main component was well preserved, despite the pre-washing process (Figure 1).

Each replication represents an average of five biological replications. Average thickness and average FW of separated apple peel were 0.8 ± 0.5 mm and 21.7 ± 10.2 g. As shown in Table 1, the weight of the peel accounted for 9.3% of the whole apple (peel + flesh). The water contents of each tissue were 80 ± 0.7% in peel and 85.4 ± 0.7% in flesh. The peel and flesh were separated in order to verify the accuracy of the peel to flesh ratio. In all experiments, the extraction of health promoting compounds was performed on separated tissues: peel and flesh. Moreover, peel and flesh samples were summed by their calculated proportions to represent the whole apple (peel + flesh) sample. Therefore, in all examination, comparison of all samples which whole apple (peel + flesh), only flesh or peel was accessed. In addition, to determine the actual effects of pre-washed apple, the dry weight value was converted into the fresh weight 100 g value according to the water contents of each portion. Apple is known to be rich in vitamin C, fiber, sugar, phenolic compounds and flavonoids [27]. Generally, these antioxidants are more present in the peel than in the flesh [28]. However, due to the problem of residual pesticides and bacteria that cause food poisoning, apples are normally washed multiple times, or the peel is removed before eating. Pre-washing apples can be an alternative way to resolve this problem, thus not only reducing the inconvenience of peeling the apple, but also allowing the intake of antioxidants [29]. As a result, consuming pre-washed apples which contained peel will increase the health promoting consumption of fresh fruits. This will affect the daily recommended fruit and vegetable intake, depending on the variety and size of the apple.

### 3.2. Total Antioxidant Activity and Antioxidant Compounds Determination

Using the kind of pre-washed apple used for fresh-cut fruit, which was washed via the ozonized water washing process, the proportion of antioxidants in the peel in relation to the whole pre-washed apple was examined. The antioxidant activities were expressed via a comparison between whole apple (peel + flesh) and flesh (Figure 1). The result was shown to be significantly different (*p* < 0.05) by Student *t*-test (*n* = 3). DPPH and FRAP assays were performed to determine antioxidant activity. The whole apple (peel + flesh) showed higher antioxidant activity, with 37.33 ± 2.49 mg GAE∙100 g^−1^ FW according to DPPH, and 24.09 ± 4.25 mg GAE∙100 g^−1^ FW according to FRAP. These antioxidant activities could be explained by the antioxidant contents. According to the TPC and TFC assays, the whole apple (peel + flesh) antioxidant contents were 40.27 ± 1.18 mg GAE∙100 g^−1^ and 0.97 ± 0.14 mg NAE∙100 g^−1^, respectively. According to this result, consumption of the whole apple (peel + flesh) is expected to offer more antioxidants than consuming only the flesh. This greater antioxidant content in the whole apple (peel + flesh) than in the same quantity of flesh alone suggests that the antioxidant contents of the peel are more effective than those in the flesh. Studies on antioxidants in fresh food have been focused on reducing specific diseases, such as cancer, caused by oxidation stress [30]. A previous study found that antioxidants can scavenge free radicals, so health might be improved by increasing antioxidant intake due to the neutralizing nature of antioxidant activity [31,32,33]. Hence, the higher antioxidant intakes derived from fresh food, such as apples, are also correlated with antioxidant activity in the body [34,35,36]. Previous research conducted by Wojdyło et al. [37] found that among the different types of apple cultivars, most bioactive compounds are in the apple peel, meaning the whole apple (peel + flesh) has a higher antioxidant capacity than the same amount of apple flesh alone. On the other hand, in a study comparing the consumption habits of peeled apples versus apples with peel, the former was found to have a roughly 2–10-fold higher antioxidant activity and antioxidant content than the latter [38]. “Fuji” pre-washed apples showed similar results (Figure 1). The antioxidant activity of the peel was about 4~5 times higher than the same amount of flesh, according to DPPH radical scavenging activity assays. Apple antioxidants is believed to be mostly attributed by apple peel, which is mainly consist of total phenolic contents, flavonoids, and triterpenoids [39,40]. In whole apple (peel + flesh), 100 g FW, 41% of the total flavonoid contents, and 31% of the phenolic contents were contributed by the peel in this experiment.

### 3.3. Effect of Apple Containing Triterpenoids on Cellular Lipid Droplets

**Figure 2:**
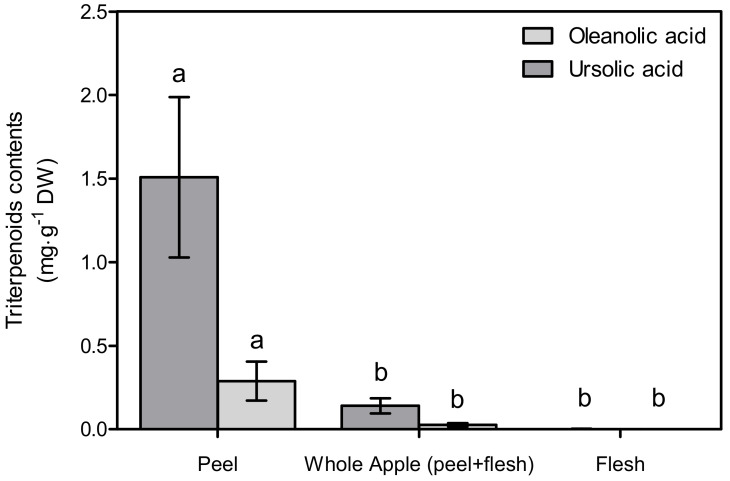
Triterpenoid contents of pre-washed apple peel, whole apple, and flesh. Different letters indicate significant differences (*p* < 0.05) among the tissues by Tukey’s HSD test (*n* = 3). Whole apple bioactive contents were calculated per peel and flesh portion.

In the peel, the ursolic acid and oleanolic acid contents were 1.51 ± 0.48 mg∙g^−1^ DW and 0.29 ± 0.12 mg∙g^−1^ DW, respectively, while in the whole apple (peel + flesh), they were 0.14 ± 0.04 mg∙g^−1^ DW and 0.03 ± 0.01 mg∙g^−1^ DW (Figure 2). These values are significantly different (*p* < 0.05) compared to the flesh, in which nothing has been detected. Likewise, the concentrated extract used on the cell culture mirrored these values. According to the DMSO, 1 g DW∙mL^−1^ of peel, the whole apple, and the concentrated flesh extract contained 3.30 ± 1.05 mM, 0.31 ± 0.09 mM and 0.00 ± 0.00 mM of ursolic acids, respectively.

**Figure 3:**
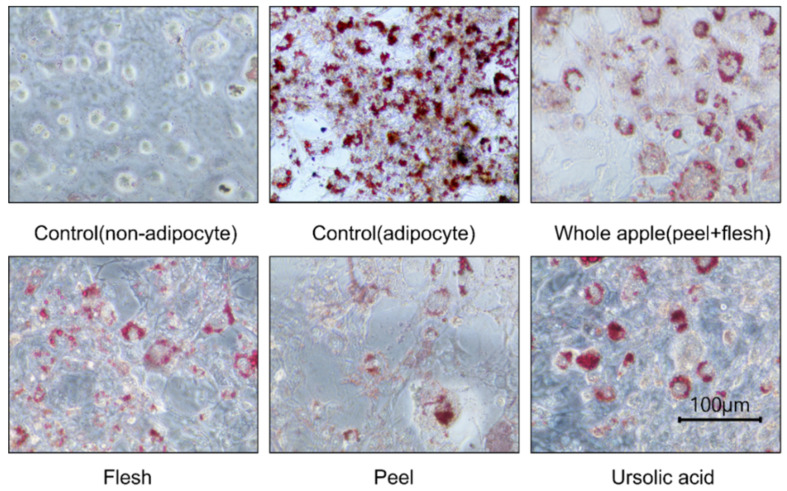
3T3-L1 cell adipocyte images, observed and photographed using a microscope (×400) after cellular lipid accumulation was stained with Oil-Red O solution. The cells were from 70% methanol-treated extracts (1 μg dry powder in 1 mL of DMSO) of peel, flesh, and whole apple (peel + flesh) concentrated samples, or those treated with ursolic acid 5 μM. During the three repetitions of the treatments, a representative photograph was taken.

The ursolic acid concentration in each sample also affected the results of the cell culture experiments. Nine days after differentiation, the control (adipocytes and non-adipocytes) and sample extract treatment groups were stained in the Oil-Red O assay (Figure 3). The red parts show where the stained cellular lipids accumulated. The less red-stained parts show areas with less lipid accumulation in the cell. Therefore, in the cell photograph, low lipid accumulation can be observed in the peel extract, or when ursolic acid was applied.

Moreover, the peel extract treatment groups show similar levels to the ursolic acid 5 μM treatment groups. On the other hand, the apple flesh extract treatment group shows no reduction in fat accumulation and was not very different from the control group. The whole apple (peel + flesh) treatment showed reduced lipid accumulation based on the values, but this was not significantly different. Ursolic acid and oleanolic acid were specifically found in the apple peel. The significantly higher levels of health-promoting compounds found in the whole apple (peel + flesh) are due to the peel. This result implies that considerable amounts of antioxidants, and maybe even all triterpenoids, would be removed if the apple was peeled. As naturally sourced ursolic acid has recently been explored for its key anti-obesity benefits, apple peel organic extracts have shown lipid suppression. Furthermore, since each apple variety has a different content of ursolic acid, if apples with high ursolic acid content were used in pre-washed fresh-cut fruit, it would be possible to consume enough ursolic acid to suppress lipids, even with lower apple intake [41].

### 3.4. 3T3-L1 Cell Lipid and Fatty Acid Suppression Effect by Different Apple Tissue Extracts

**Figure 4:**
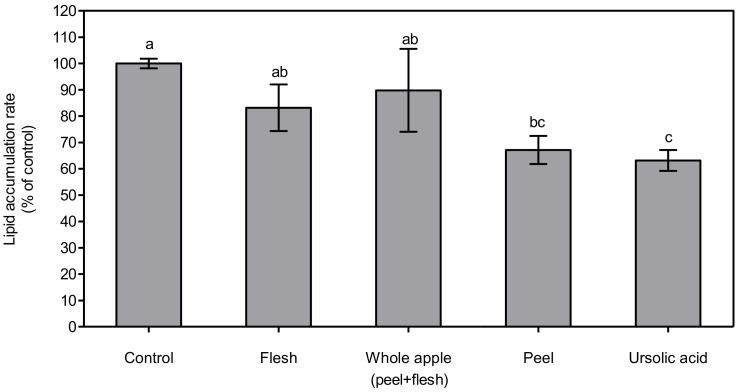
Lipid accumulation rate determined by Oil-Red O assay in 3T3-L1 cells treated with 70% methanol extract (1 μg dry powder in 1 mL of DMSO) from peel, flesh, and whole apple (peel + flesh) samples, or ursolic acid 5 μM-treated samples. The data are represented as % of lipid accumulation compared to control. The bar and error bar indicate mean and standard deviation (*n* = 3). Different letters indicate significant differences among the groups according to Tukey’s HSD test at *p* < 0.05.

The lipid accumulation rate in 3T3-L1 cells was measured via Oil-Red O assay, and the cellular lipid concentration in differentiated 3T3-L1 cells was determined by a triglyceride analysis kit. After the extraction of the Oil-Red-stained O parts, the absorbance was expressed as % of control (Figure 4). In this experiment, the ursolic acid in the apple peel reduced the lipid droplet in a similar way to previous studies. Hence, the apple peel extracts in this experiment only contained non-polar compounds, including ursolic acid, fatty acids, and long-chain waxes, and did not contain polar compounds, including polyphenolic compounds and vitamins, which potentially suppress lipid accumulation [42]. The degree of the suppression of lipid accumulation by apple peel treatment may be even higher than was observed in this experiment. This result suggests that the consumption of pre-washed apples, including their peel, potentially reduce lipid accumulation in the human body.

**Figure 5:**
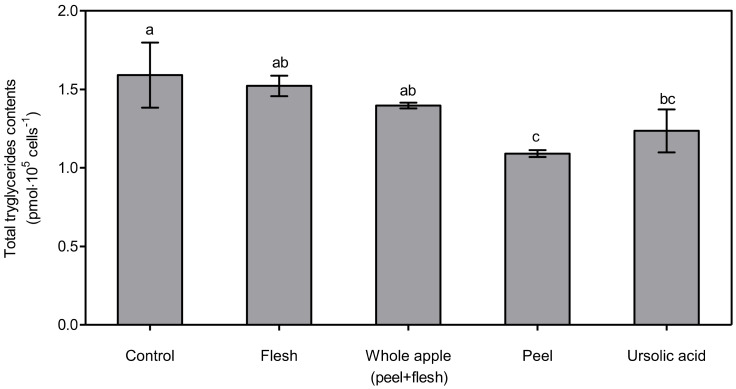
Total triglyceride content determination from 3T3-L1 cells treated with 70% methanol extracts (1 μg dry powder in 1 mL of DMSO) from peel, flesh, and whole apple (peel + flesh) samples or ursolic acid 5 μM treated samples. The data represent the mean and standard deviation (*n* = 3). Different letters indicate significant differences among the groups according to Tukey’s HSD test at *p* < 0.05.

The lowest total triglyceride content was observed in the ursolic acid-treated group, at 63.18 ± 4.00%, which was significantly lower (*p* < 0.05) than in the control, flesh, and whole apple (peel + flesh) groups. The next lowest total triglyceride content was found in the treated peel group, at 67.19 ± 5.34% of control. The treated peel group showed significantly lower (*p* < 0.05) content than the control and flesh groups, but not the whole apple (peel + flesh) group. This tendency was also found via cellular lipid accumulation assay, the results of which are shown in Figure 4. The total triglyceride content is expressed as pmol∙10^5^ cells^−1^ (Figure 5). The total triglyceride contents in the cells were high, and manifested in the order of flesh, whole apple (peel + flesh), ursolic acid, and peel treatment groups. The peel treatment groups (1.09 ± 0.02 pmol∙10^5^ cells^−1^), in particular, showed a significantly low (*p* < 0.05) total triglyceride content compared with the control (1.59 ± 0.21 pmol∙10^5^ cells^−1^) and flesh treatment groups (1.52 ± 0.06 pmol∙10^5^ cells^−1^). Considering two different cellular lipid measurement results, the apple peel extract showed strong lipid suppression ability. The whole apple (peel + flesh) treatment reduced the total triglyceride content possibly due to the triterpenoids in apple peel, but this value was not significantly different (*p* < 0.05) compared to the control.

This result is also consistent with the previous report that apple could have anti-obesity effects due to the ursolic acid in the waxy coating of the peel [43]. According to the study by Katashima et al. [44], a mouse group treated with ursolic acid showed reduced body mass gain, serum leptin, insulin, and glucose levels, with improvements in insulin resistance and metabolic disorders. In addition, it has been found that the transcription factors involved in lipid metabolism, such as peroxisome proliferator-activated receptor gamma (PPARγ), were attenuated in 3T3-L1 adipogenic cells after treatment with 2.5 μM to 1 μM ursolic acid [45]. Moreover, there was an increase in the AMP-activated protein kinase (AMPK) level resulting from the inhibition of liver kinase B1, suggesting the potential anti-obesity effect of ursolic acid [46,47]. Therefore, apples that include peel are also expected to show the same mechanisms as described above. The final concentrations of ursolic acid in the peel and whole apple samples according to the lipid suppression, TG quantification, and fatty acid quantification assays, using 3T3-L1 cells, were 3.30 ± 1.05 µM and 0.31 ± 0.09 µM, respectively. Corresponding with this data, the lipid suppression effect in the three tissues was increased depending on the amount of ursolic acid present in the peel. The flesh sample, due to the absence of apple peel, did not suppress the lipid droplet in 3T3-L1 pre-adipocytes compared with the peel and whole apple treatments. The lipid suppression effects of ursolic acid were also shown in previous research [6,39,45]. It has been reported that ursolic acid concentration varies depending on apple cultivar and storage conditions [48]. These comprehensive studies on apple peel and ursolic acid imply that apple peel is an excellent source for ursolic acid [49].

### 3.5. 3T3-L1 Cellular Fatty Acid Determination

To analyse the type of FA present in cells, the lipids of the entire cell were extracted and then methyl-esterified for analysis with GC-MS [17]. Each FA was quantified as a methyl heptadecanoic acid equivalent concentration (Table 2). The table was constructed based on saturated fatty acids (SFA), which were found to have high concentrations in cells, while the summed monounsaturated fatty acid (MUFA), polyunsaturated (PUFA) concentration and the MUFA/SFA value are also represented (*n* = 3). The total FA contents of treated peel extract cells and ursolic acid-treated cells were significantly reduced (*p* < 0.05) compared to the control cells. Likewise, previous research on the fatty acid synthase inhibitory effect of ursolic acid has reported that the IC_50_ value of fatty acids synthase inhibition of ursolic acid is 6.0 μg·mL^−1^ [50]. Among the FAs, the C16:0 concentration was the highest in all the groups. In the apple peel treatment group and ursolic acid treatment group, the C16:0 concentrations were significantly reduced (*p* < 0.05). The C16:0 of palmitic acid represents 20–30% of the total FA in the membrane phospholipid and fatty triacylglycerol [51]. These results imply that peel treatments have the same anti-obesity effects as ursolic acid treatments on 3T3-L1 cells.

AMPK activated by ursolic acid stimulates fatty acid oxidation and regulates fatty acid synthase (FAS) [52,53]. Moreover, the treatment groups of 1 mg DW∙mL^−1^ peel and 5 μM of ursolic acid showed significantly reduced (*p* < 0.05) cellular SFA and MUFA, but not PUFA. Compared to the flesh and peel treatment, the peel treatment group showed a significantly reduced (*p* < 0.05) SFA concentration. The treatment of the peel significantly reduced the ratio of C16:1/C16:0 to 76% in the control cells (*p* < 0.05).

## 4. Conclusions

Only the apple peel containing ursolic acid showed reduced lipids in the 3T3-L1 cell. This experimental result suggests that whole apple consumption may potentially reduce the cellular lipid level in 3T3-L1 cells, and could be a good source of antioxidants, compared with eating only the apple flesh. In conclusion, consuming pre-washed apples could contribute to human health, due to their anti-obesity effect. Although the portion of apple peel in the whole apple accounts for less than 10% of the apple’s flesh, eating the peel allows consumers to take in higher concentrations of health-promoting compounds.

## Figures and Tables

**Table 1:** Morphological characteristics of ‘Fuji’ pre-washed apple (*Malus pumila* Mill).

	Pre-Washed Apple (100%) ^z^	
	Flesh (90.7%)	Peel (9.3%)
Fresh weight (g)	212.4 ± 8.2	21.7 ± 10.2
Dry weight (g)	31.0 ± 1.2	4.3 ± 2.0
Moist content (%)	85.4 ± 0.7	80.0 ± 0.7

**^z^** The pre-washed ‘Fuji’ (*Malus pumila* Mill) apple was bought from the local market. Each apple was peeled uniformly using Jonny Apple Peeler. All values were represented as the means ± standard deviation of pre-washed apples.

**Table 2:** Quantified fatty acid concentrations of 3T3-L1 cells after treatment with 70% methanol (1 mg dry powder in 1 mL of DMSO) for extracts of “Fuji” pre-washed apple (*Malus pumila* Mill) or ursolic acid 5 μM.

Fatty Acids Concentration (μg·10^5^ Cells^−1^)		1 mg DW·mL^−1^			5 μM
	Control	Whole Apple (Peel + Flesh)	Flesh	Peel	Ursolic Acid
C16:0	11.97 ± 0.60a	10.61 ± 1.52ab	12.15 ± 1.05a	8.09 ± 1.40b	7.53 ± 2.08b
C18:0	5.49 ± 0.67a	4.84 ± 1.33a	5.17 ± 1.25a	3.25 ± 0.72a	3.48 ± 0.68a
C18:1n-9 (c)	3.88 ± 0.24a	3.13 ± 0.83b	3.37 ± 0.74a	2.07 ± 0.48b	2.41 ± 0.49a
C16:1	2.64 ± 0.20a	1.86 ± 0.29bc	2.17 ± 0.18ab	1.34 ± 0.16d	1.47 ± 0.28cd
C14:0	1.35 ± 0.12a	0.97 ± 0.16ab	1.13 ± 0.17a	0.70 ± 0.12b	0.71 ± 0.18b
C16:1/C16:0	0.22 ± 0.01a	0.18 ± 0.01a	0.18 ± 0.00a	0.17 ± 0.02b	0.20 ± 0.03a
C18:1/C18:0	0.71 ± 0.05a	0.65 ± 0.01a	0.65 ± 0.02a	0.64 ± 0.04b	0.69 ± 0.00a
MUFA/SFA	0.40 ± 0.00a	0.35 ± 0.03a	0.35 ± 0.03a	0.33 ± 0.05a	0.39 ± 0.04a
Total SFA^z^	19.08 ± 1.19a	16.66 ± 2.97ab	18.70 ± 2.27a	12.20 ± 2.00c	11.90 ± 2.97bc
Total MUFA^y^	7.59 ± 0.50a	5.91 ± 1.43a	6.53 ± 1.12a	4.05 ± 0.76b	4.58 ± 0.87b
Total PFA^x^	1.01 ± 0.16a	0.89 ± 0.33a	0.92 ± 0.33a	0.57 ± 0.19a	0.70 ± 0.18a
Total FA	27.77 ± 1.83a	23.54 ± 4.74a	26.23 ± 3.69a	16.86 ± 2.73b	17.25 ± 4.02b

The five highest concentration of FAs were represented by order. ^z^ include C16:0 C18:0, C14:0, C24:0, C22:0, C10:0, C12:0. ^y^ include C18:1n-9(t), C14:1, C18:1n-9(c), C16:1. ^x^ include C18:2n-6(t), C18:3n-6, C20:2, C20:3n-6, C22:6n-3. C20:4n-6, C20:3n-3, C20:4n-6. Total FA include z, y, x. All value were represented ± standard deviation, (*n* = 3) and the different letters indicate significant differences (*p* < 0.05) according to Tukey’s HSD tests.

## Data Availability

Data is contained within the article.
